# Early detection of steroid-induced femoral head necrosis using ^99m^Tc-Cys-Annexin V-based apoptosis imaging in a rabbit model

**DOI:** 10.1186/s10020-020-00248-1

**Published:** 2020-12-03

**Authors:** Xiaolong Wang, Jianbo Li, Da Man, Rui Liu, Jianmin Zhao

**Affiliations:** 1 Department of Hand and Foot Microsurgery, Second Affiliated Hospital of Inner Mongolia Medical University, No. 1 Yingfang Road, Hohhot, 010030 China; 2 Department of Nuclear Medicine, Inner Mongolia Medical University Affiliated Hospital, No. 1 Tongdao North Street, Hohhot, 010050 China; 3 Key Laboratory of Molecular Imaging, Inner Mongolia Autonomous Region, No. 1 Tongdao North Street, Hohhot, 010050 China; 4 Department of Orthopaedics, Affiliated Hospital of Inner Mongolia Medical University, No. 1 Tongdao North Street, Hohhot, 010050 China

**Keywords:** ^99m^Tc-Cys-Annexin V, SPECT imaging, MRI, Steroid-induced femoral head necrosis

## Abstract

**Background:**

At present, the early diagnosis of femoral head necrosis mainly relies on Magnetic resonance imaging (MRI), and most early patients are difficult to make an accurate diagnosis. Therefore, to investigate the early diagnostic value of ^99m^Tc-Cys-Annexin V Single-photon emission computed tomography (SPECT) imaging were compared with MRI in rabbit models of steroid-induced femoral head necrosis.

**Methods:**

The animal model of steroid-induced femoral head necrosis (SIFHN) was established in 5-month-old healthy New Zealand white rabbits by injecting horse serum into ear vein and methylprednisolone into gluteal muscle, the purpose of modeling is to simulate the actual clinical situation of SIFNH. ^99m^Tc-Cys-Annexin V SPECT imaging and MRI were performed at 2nd week, 4th week, and 6th week after modeling. After that, histopathology was used to verify the success of modeling. Apoptosis was detected by transmission electron microscopy (TEM) and terminal deoxynucleotidyl transferase-mediated dUTP-biotin nick end labeling assay (TUNEL).

**Results:**

At 2 weeks after the injection of hormone, ^99m^Tc-Cys-Annexin V SPECT image showed abnormal radioactive uptake in the bilateral femoral head. And over time, the radioactivity concentration was more obvious, and the ratio of T/NT (target tissue/non-target tissues, which is the ratio of femoral head and the ipsilateral femoral shaft) was gradually increased. In the ^99m^Tc-Cys-Annexin V SPECT imaging at each time point, T/NT ratio of the model group was significantly higher than that of the control group (P < 0.01); at 4 weeks after the injection of hormone, MRI showed an abnormal signal of osteonecrosis. At 2, 4, and 6 weeks after hormone injection, apoptosis was observed by TUNEL and TEM.

**Conclusions:**

^99m^Tc-Cys-Annexin V SPECT imaging can diagnose steroid-induced femoral head necrosis earlier than MRI, and has potential application value for non-invasively detecting early and even ultra-early stage of femoral head necrosis.

## Background

Glucocorticoid is widely used in the treatment of systemic lupus erythematosus (Wang et al. 2013; Kuroda et al. 2015), leukemia (Vogel et al. 2010), organ transplantation (Zhang et al. 2017), malignant tumors and other diseases, which will lead to an increase in the incidence of femoral head necrosis. Steroid-induced femoral head necrosis (SIFHN), which has become the most common cause of non-traumatic femoral head necrosis, seriously affects patients' quality of life and labor capacity, and brings enormous burden to society (Wu et al. 2015).

At present, for the diagnosis of femoral head necrosis, most experts use the staging standard of Association research circulation osseous (ARCO) (Gardeniers 1992), which was established in 1992. ARCO staging divides femoral head necrosis into stage 0–IV. Stage 0: The pathological biopsy results are consistent with the of the femoral head necrosis. The rest of the examinations are normal, and there are generally no clinical symptoms. This situation is difficult to find in clinical practice. Stage I: The disease in this stage cannot be diagnosed by X-ray and Computed tomography (CT). The Emission computed tomography (ECT) and/or Magnetic resonance imaging (MRI) examination is positive. Only a small number of patients may have clinical symptoms. Stage I is the early stage of femoral head necrosis, which is difficult to diagnose clinically. Stages II–IV: The diseases in these stages are relatively easy to diagnose clinically. At present, the early diagnosis of femoral head necrosis mainly relies on MRI (Karantanas and Drakonaki 2011; Manenti et al. 2015), which can show abnormal reactions such as edema, necrosis and bone marrow repair in bone marrow tissues (Shapiro et al. 2009; Yamaguchi et al. 2011; Arbab and König 2016). The pathological changes of femoral head necrosis can also be judged according to the different imaging changes of MRI (Shapiro et al. 2009; Yamaguchi et al. 2011). Therefore, MRI is considered to be a gold standard for non-invasive and early diagnosis of osteonecrosis (Manenti et al. 2015; Arbab and König 2016; Liu et al. 2017). However, MRI is unable to diagnose the femoral head necrosis in stage 0. If the femoral head necrosis in stage 0 can be diagnosed early, active treatment measures can be taken to protect joint function and avoid joint replacement (Arbab and König 2016; Liu et al. 2017; Chan and Mok 2012).

In recent years, more and more studies have shown that SIFHN is closely related to bone cell apoptosis (Youm et al. 2010; Mutijima et al. 2014; Bai et al. 2016), and apoptosis occurs in the early stage of femoral head necrosis and throughout the necrosis process. Therefore, based on the relationship between SIFHN and apoptosis, we wanted to use the detection of bone cell apoptosis to achieve the purpose of diagnosing early SIFHN. Because both apoptosis and necrosis can make terminal deoxynucleotidyl transferase-mediated dUTP-biotin nick end labeling assay (TUNEL) label positive, which leads to a false positive rate of apoptotic cells detected by TUNEL, while transmission electron microscopy (TEM) is considered as the gold standard method to identify apoptotic cells. Therefore, in order to eliminate the interference of cell necrosis, we further use TEM to effectively distinguish between apoptotic and necrotic cells.

The molecular probe ^99m^Tc-Cys-Annexin V (Lu et al. 2013), which has similar biological characteristics to the physiological physiology of Annexin V, is introduced into the model animals and specifically binds to phosphatidylserine on the cell membrane of apoptotic cells (Engeland et al. 1998). Single-photon emission computed tomography (SPECT) is used to detect the distribution of molecular probe in vivo, and to achieve the purpose of non-invasive dynamic detection of apoptosis (Me et al. 2012). We found that ^99m^Tc-Cys-Annexin V SPECT imaging could detect rabbit SIFHN earlier than ^99m^Tc-MDP bone imaging in the previous study (Wang et al. 2016), but could ^99m^Tc-Cys-Annexin V SPECT imaging detect femoral head necrosis earlier than MRI?

In this study, ^99m^Tc-Cys-Annexin V was used to detect the apoptosis of bone cells in rabbit model of steroid-induced femoral head necrosis. Compared SPECT imaging results with MRI results, ^99m^Tc-Cys-Annexin V SPECT imaging could be proved to diagnose SIFHN earlier than MRI imaging.

## Methods

### General

Horse serum was purchased from HyClone. Methylprednisolone was purchased from Pfizer Pharmaceuticals (Belgium). Penicillin injection was purchased from Harbin Pharmaceutical Group Holding Co. Sodium pentobarbital was purchased from Merk, Germany. The TUNEL kit was purchased from Roche, USA. Hematoxylin and eosin were purchased from Amresco, USA. DAPI were purchased from Themo, USA. Fluorescent sealants were purchased from Biyuntian Company, China.

Ultra-thin slicer: Leica EM UC6. Optical microscope (model 107JC): Shanghai Precision Instrument Factory. Fluorescence microscope: Nikon, Tokyo, Japan. Transmission electron microscope: FEI Tecnai Spirit. Magnetic resonance scanner: Signal 3.0 T MRI produced by GE, USA. SPECT/CT scanner: Discovery NM/CT 670 SPECT/CT from United States GE Company.

### Experimental animal

The animals used in this experiment strictly complied with the “Guidelines for the Care and Use of Laboratory Animals” formulated by Inner Mongolia Autonomous Region, China, and approved by the Institutional Animal Care and Use Committee of Inner Mongolia Medical University (No.: 2013037).

New Zealand white rabbits (~ 5 months old, 2.65 ± 0.21 kg, male or female, n = 54) were purchased from Xi'an Dilepu Biological Resources Development Co., Ltd. (Qualification No.: SCXK (Shan) 2014-001). The experimental rabbits were given standard rabbit pellet feed and free drinking tap water, single cage feeding, full light and good ventilation.

### The establishment of model animals

*Grouping* After 1 week of adaptive feeding, 24 of New Zealand white rabbits (n = 54) were assigned to the control group by random number table method, and the remaining 30 were assigned to the model group.

*Model establishment* In the model group, model rabbits were established by using hormone combined with allogeneic serum. Briefly, a rabbit was injected with horse serum (10 ml/kg) via ear vein at the first time. After 2 weeks, the same rabbit was injected with horse serum (5 ml/kg) every day for 2 days. After 2 weeks, methylprednisolone acetate (7.5 mg/kg) was injected at gluteal muscle, and injected once every 3 days for 6 weeks. In the control group, the same amount of physiological saline was intravenously injected through the ear vein of the rabbits twice a week for 6 weeks. During the modeling period, two groups of rabbits were intramuscularly injected with 800,000 units of penicillin, 2 times/week for 12 times.

### Imaging protocol for experimental rabbit

At 1 week before modeling and 2, 4, and 6 weeks after hormone injection, 10 rabbits from the model group and 8 rabbits from the control group were randomly selected to perform MRI imaging and ^99m^Tc-Cys-Annexin V SPECT imaging. The rabbits were anesthetized with a 3% sodium pentobarbital solution (25–30 mg/Kg, Merk, Germany). The anesthetized rabbit was placed in the supine position on the orthopedic support frame to perform MRI imaging. After 2 days, the model rabbit was fixed at the same position to perform ^99m^Tc-Cys-Annexin V SPECT imaging.

### MRI

MRI (American GE 3.0 T MRI) was used to scan the bilateral femoral head of the rabbits. The scanning sequence was T1WI, T2WI and fat suppression imaging was added. The parameters for MRI were: coronal T1WI scan parameters (TR/TE: 900/18.6 ms and matrix: 384 × 256), coronal T2WI scan parameters (TR/TE: 3400/85 ms, matrix: 320 × 256), fat suppression sequence (TR/ TE: 1456/70 ms), layer thickness: 3 mm, layer spacing: 3 mm, field of view: 18 cm, scan time 10–15 min.

The positive criteria for MRI diagnosis of femoral head necrosis: T1WI showed a point, thin line, flaky low signal, and (or) T2WI showed a point, thin line, flaky low signal or high signal. Joint effusion is considered an indirect sign.

### SPECT imaging

According to the literature (Lu et al. 2013), ^99m^Tc-Cys-Annexin V was prepared by direct reduction labeling method, and the radiochemical purity was over 95% by Radio-HPLC. ^99m^Tc-Cys-Annexin V (18.5 MBq for each rabbit) was injected through rabbit ear vein. At 1 h after injection, the anesthetized rabbits were placed on a SPECT scanner for SPECT imaging. Image acquisition conditions: Planar static acquisition, acquisition time (6 min/each), magnification (1.0), energy peak (140 kV), window width (20), and matrix (256 × 256). Before SPECT imaging, the model rabbits were banned from water for 8 h, and a catheter was used to urinate before imaging to avoid false positive results due to bladder filling.

*Image processing and analysis* SPECT image processing was performed using a Xeleris post-processing system workstation. A combination of qualitative and quantitative methods was used as the diagnostic criteria. (1) Qualitative method: Abnormal radioactive concentration, sparseness and defects in the bilateral femoral head were abnormal. (2) Semi-quantitative method: Region of interest (ROI) of the femoral head and the ipsilateral femoral shaft (ie, target tissue and non-target tissue) were delineated. The semi-quantitative analysis was used to determine the radioactivity counts of target tissue and non-target tissue (T/NT), and the T/NT ratio was calculated.

### Specimen collection and processing

At the end of the last time point, the model rabbits were sacrificed by injection of excess anesthetic (3% sodium pentobarbital), and the bilateral femoral heads were taken and cut along the coronal plane. Specimens for Hematoxylin–Eosin (HE) staining and terminal deoxynucleotidyl transferase (TdT)-mediated dUTP nick end labeling (TUNEL) assay were fixed in 10% formaldehyde solution and specimens for transmission electron microscopy (TEM) were fixed in 2.5% glutaraldehyde solution.

### HE staining

After 48 h, the femoral head was removed from the fixative and placed in 10% Ethylenediaminetetraacetic acid (EDTA) solution for decalcification for 2 months. After successful decalcification, the femoral head was rinsed in the water for 24 h, and then were dehydrated step by step, transparent with xylene, embedded in paraffin, and sliced (thickness 4 μm). HE staining was performed. Morphological changes of trabecular bone, bone cells, fat cells in bone marrow, hematopoietic cells, etc. were observed under light microscope.

Femoral head necrosis was determined according to the rate of empty bone lacuna: 10 fields were randomly selected, 50 bone lacuna were counted in each field, the number of empty bone lacuna was counted, and the rate of empty lacuna was calculated = (number of empty bone lacuna / 50).

### TUNEL assay

The experimental procedure was carried out in accordance with the instructions. Paraffin sections were washed with xylene for 5 min × 2, washed with gradient alcohol (100, 95, 90, 80, 70%) for 3 min × 1, rinsed with PBS × 2, treated with Proteinase K working solution at room temperature for 15–30 min and rinsed with PBS × 3. After dried, the slides were added 50 μL of TUNEL reaction mixture (pre-configuration before use), incubated at 37 °C for 1 h in the dark, and then rinsed with PBS × 3. Finally, the slices were stained with DAPI solution, and examined under a fluorescent microscope. The cells, of which the nuclei showed green fluorescence under the microscopy, were TUNEL-positive apoptotic cells. Ten fields of view were randomly selected, 50 bone cells were counted in each field, the number of apoptotic cells was counted, and Apoptosis index (AI) (number of apoptotic cells/50) was calculated.

### TEM

The decalcified specimen was cut into small bone pieces of about 1 mm3, rinsed with PBS for 15 min × 3, fixed at 1% citrate for 1 h, washed with PBS for 15 min × 3, dehydrated in gradient alcohol at room temperature for 30 min, soaked in epoxy resin embedding solution (1:1) for 2 h, polymerized in oven at 45 °C for 12 h and then in oven at 65 °C for another 48 h. Ultrathin sections (thickness 70 nm) were prepared, and the ultrastructure of bone tissue was observed by transmission electron microscopy using double staining of uranium acetate and lead citrate.

### Statistical methods

SPSS23.0 software (IBM, USA) was used for data analysis. The measurement data in the observational data were described by MEAN ± SD through the normality test. The comparison between the two groups was the group t test or the corrected t test (the statistic is t). The comparison among multiple groups was a one-way analysis of variance (the statistic is F) and pairwise comparison LSD-t test (the statistic is LSD-t). *P* < 0.05 indicated that the difference was statistically significant.

## Results

### Model animal establishment

In the model group, 4 rabbits died during the modeling process. Model rabbits were assigned to the 2nd week group (n = 9), 4th week group (n = 8), and 6th week group (n = 9) for subsequent experimental studies. According to histopathology (HE staining), the incidence of femoral head necrosis was 77.78% (7/9) at 2nd week, 87.50% (7/8) at 4th week and 88.89% (8/9) at 6th week after injection of hormone in the model group, and the success rate of modeling was 84.62% (22/26). All rabbits in the control group survived and no femoral head necrosis occurred.

### MRI

**Fig. 1:**
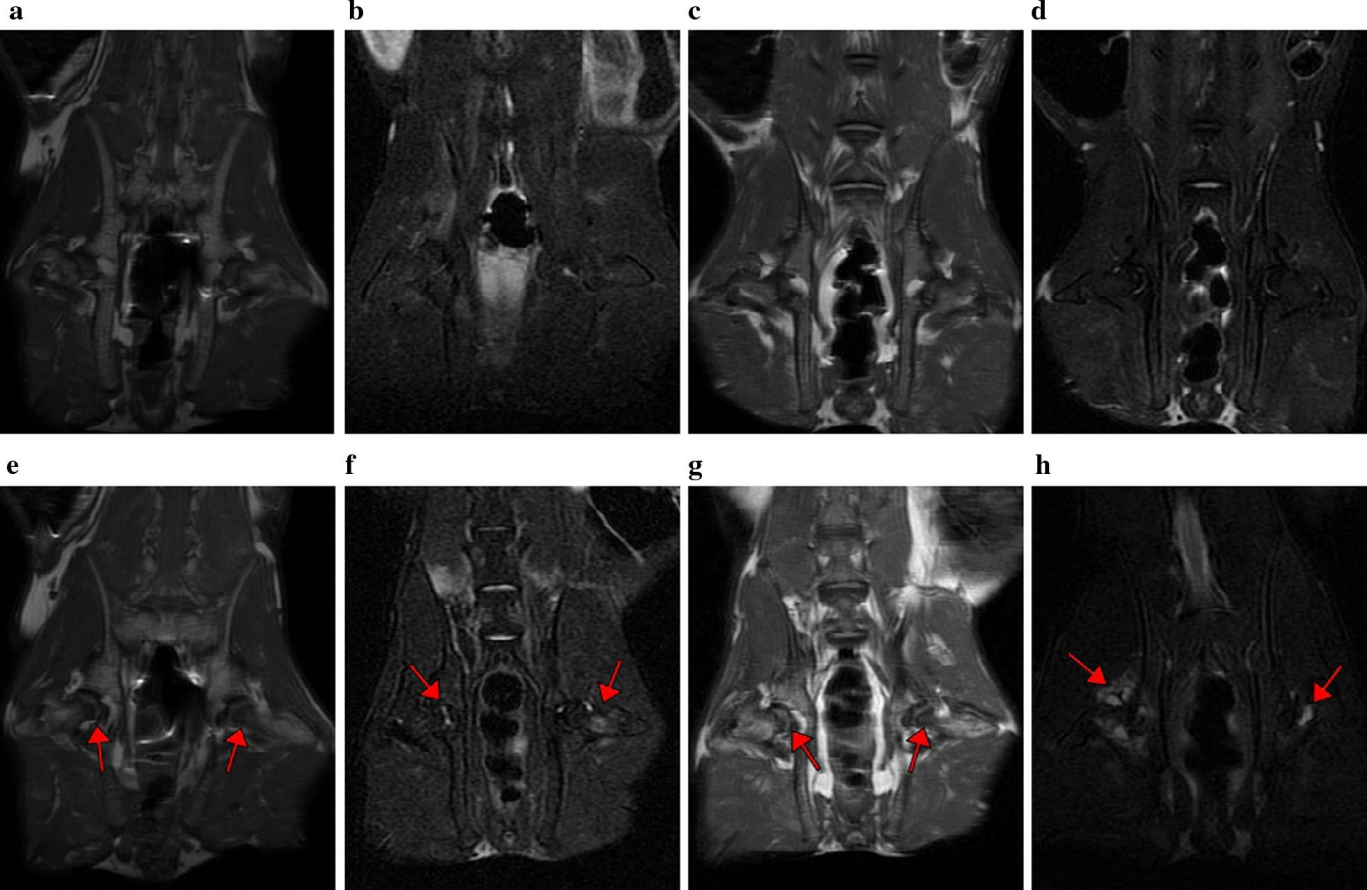
MRI. **a** and **b** were T1WI and T2WI + fat-suppression coronal image in the control group; **c** and **d** were T1WI and T2WI + fat-suppressed coronal image at 2nd week after injection of hormone in the model group; **e** and f were T1WI and T2WI + fat-suppressed coronal image at 4th week after injection of hormone in the model group; **g** and **h** were T1WI and T2WI + fat-suppressed coronal image at 6th week after injection of hormone in the model group. (Red arrows indicated osteonecrosis low/high signal in T1WI and T2WI + fat suppression sequences)

The results of the control group were shown in Fig. 1a, b: MRI T1WI (Fig. 1a) and T2WI (Fig. 1b) showed no obvious abnormal signal changes, the shape of the femoral head was normal, the joint space was normal, and there was no joint effusion. The results of the model group were shown in Fig. 1c–h: no signs of osteonecrosis were observed in the T1WI (Fig. 1c) and T2WI images (Fig. 1d) of MRI images at 2nd week. At 4th week, 5 experimental rabbits in the model group showed dot-like, thin-lined, and platelet-like low signals in TlWl (Fig. 1e), and T2WI (Fig. 1f) showed dot-like, thin-lined, and flaky high signal. At 6th week, 7 rabbits in the model group showed low signal of osteonecrosis in TlWl (Fig. 1g), and T2WI (Fig. 1h) showed high signal of osteonecrosis, and the signal of osteonecrosis was more obvious than that at 4th week. The shape of the bilateral femoral head at each time point was normal, no joint surface collapse was observed, and a small amount of joint effusion was observed.

### SPECT imaging

**Fig. 2.**
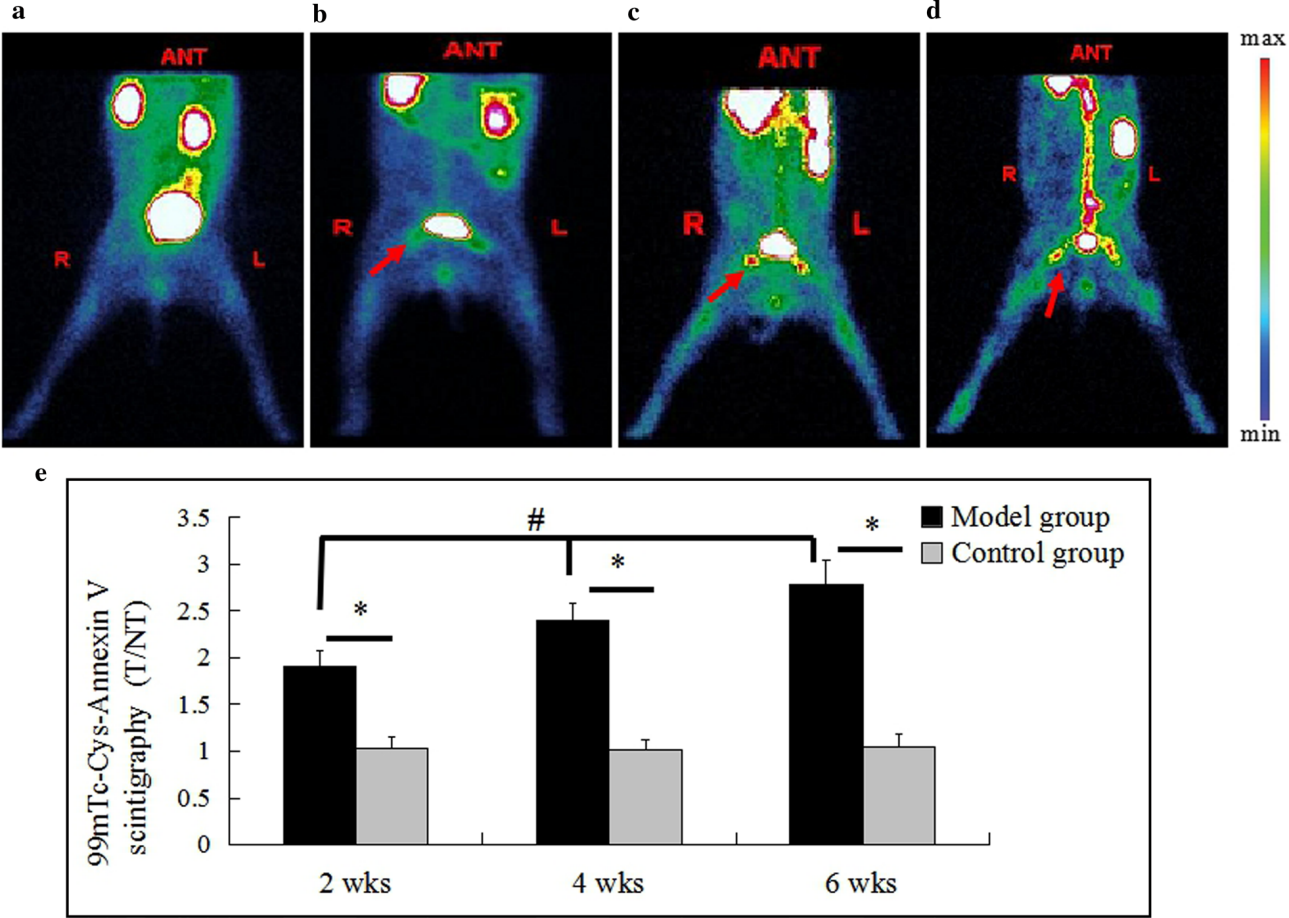
^99m^Tc-Cys-Annexin V SPECT imaging. **a** was the control group image; **b**, **c**, and **d** were the images at 2nd, 4th, and 6th week after the injection of the hormone in the model group.(Red arrows indicated abnormal radioactive concentration in the bilateral femoral heads). **e**: The T/NT ratio of the model group was significantly higher than that of the control group at each time point (* inter-group comparison, *P* < 0.05), and there was a statistically significant difference in the T/NT ratio within the model groups at each time point (# intra-group comparison, *P* < 0.05). Model group vs control group: the 2nd week (n = 9 vs 8), 4th week(n = 8 vs 8), and 6th week (n = 9 vs 8)

The results of the control group were shown in Fig. 2a that no abnormal radioactive concentration, sparseness and defect were observed in the bilateral femoral head at each time point. As shown in Fig. 2b–d, the experimental rabbits showed abnormal radioactive concentration in the bilateral femoral heads at 2nd, 4th, and 6th weeks. As the modeling time progressed, the abnormal radioactive concentration of the femoral head gradually increased. The semi-quantitative method showed that the T/NT ratio of the model group at each time point was significantly higher than that of the control group (*P* < 0.05), and there was a statistically significant difference in the T/NT ratio within the model groups at each time point (*F* = 67.947, *P* < 0.05) (see Fig. 2e).

### HE staining

**Fig. 3:**
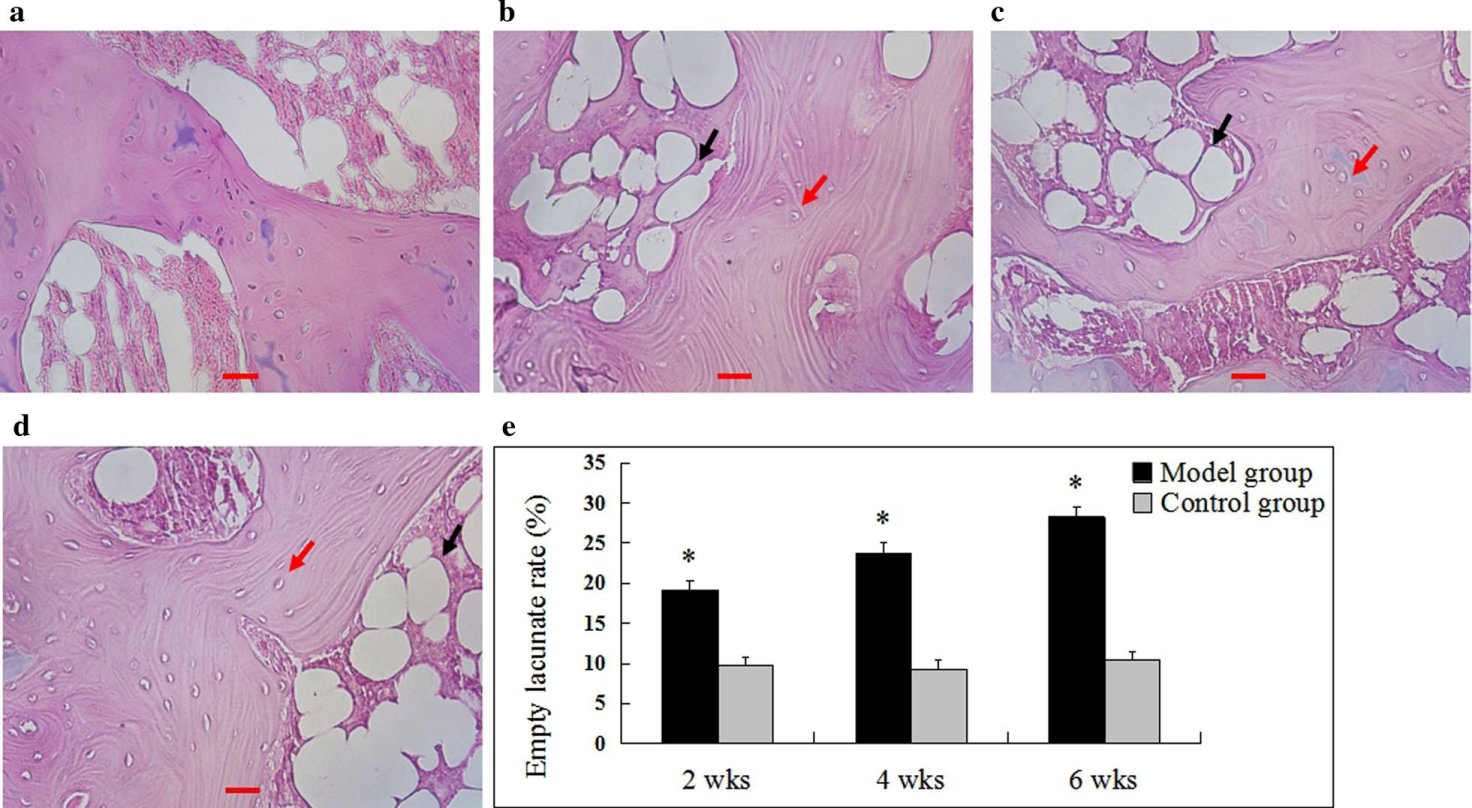
Histological images in bone tissues in both groups detected via H&E staining (× 400). No obvious osteonecrosis was observed in control group (**a**). Typical osteonecrosis of the femoral heads was observed in model group (**b**–**d**). Scale bar = 100 um. The rate of empty lacunae in model group was significantly higher than that control group at each time point (**e**) (*compared with control group, *P* < 0.05. The red arrow shows the empty lacunae, and the black arrow shows the fat cells). Model group vs control group: the 2nd week (n = 9 vs 8), 4th week (n = 8 vs 8), and 6th week (n = 9 vs 8)

The specimens of the control group showed no osteonecrosis at each time point (Fig. 3a). In the model group, at 2nd week after the injection of hormones, histopathological resulted showed typical manifestations of femoral head necrosis: bone cell pyknosis, increased number of empty bone cell lacunae, and decreased hematopoietic cells in the medullary cavity, adipocyte hyperplasia, hypertrophy, etc. (Fig. 3b). With the passage of time, osteonecrosis gradually became obvious. At 4th week, the hematopoietic cells in the bone marrow were replaced by a large number of fat cells. The fat cells in the medullary cavity increased and accumulated, and some of them merged into a blister, and the bone marrow was hemorrhagic and necrotic. The lacuna and the lacuna of the bone are vacuolated (Fig. 3c). At 6th weeks, osteonecrosis was more pronounced (Fig. 3d). The pathological changes of osteonecrosis at each time point were mainly located in the subchondral area. The rate of empty bone lacuna at 2nd, 4th, and 6th weeks after injection of the hormone in the model group was significantly higher than that of the control group (*P* < 0.05) (Fig. 3e).

### TUNEL assay

**Fig. 4:**
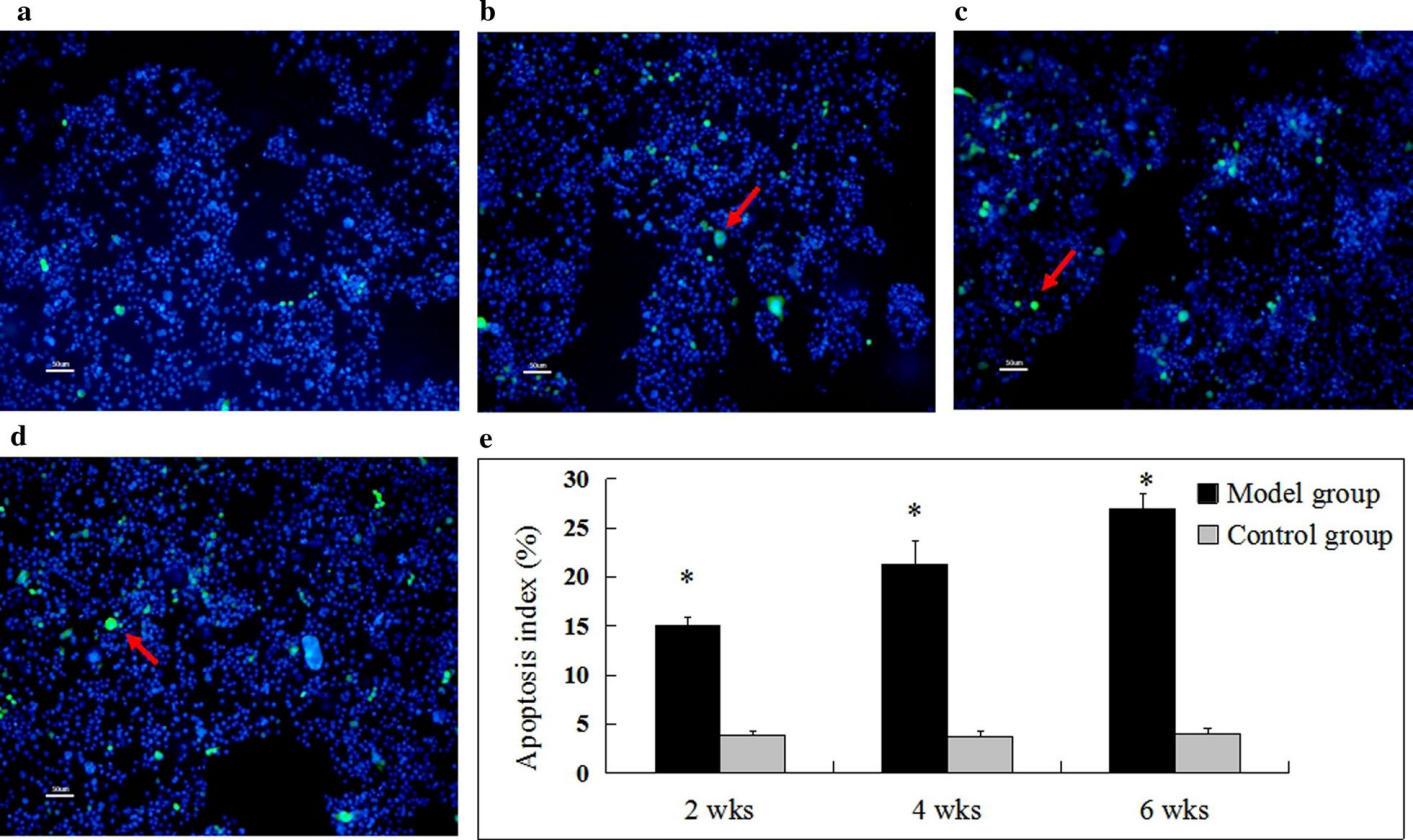
Apoptosis was detected via TUNEL assay with a fluorescence microscope (× 200). There was no abnormal apoptosis detected in control group at any of the time points (images **a**). TUNEL staining showed gradual increases in apoptosis in model group (images **b**–**d**). Scale bar = 50 um. The apoptosis index in model group was significantly higher than that in control group at each time point (images **e**). DAPI wss used to mark all cells, blue dots in the figure, and TUNEL wss used to stain apoptotic cells, green dots in the figure) (*compared with control group, *P* < 0.05. The red arrow shows the TUNEL-positive cells). Model group vs control group: the 2nd week (n = 9 vs 8), 4th week(n = 8 vs 8), and 6th week (n = 9 vs 8)

In the control group, no obvious abnormal apoptotic bone cells were observed during the whole experiment, and the number of apoptotic cells was relatively stable (Fig. 4a). The TUNEL results of the model group showed that apoptotic bone cells with nucleus emitting green appeared from 2nd week (Fig. 4b), and apoptotic bone cells gradually increased at 4th week (Fig. 4c), and the number of apoptotic bone cells increased significantly at 6th week (Fig. 4d). With the prolongation of modeling time, the number of apoptotic cells increased gradually. The apoptotic index of the model group at each time point was significantly higher than that of the control group (*P* < 0.05) (Fig. 4e).

### TEM

**Fig. 5:**
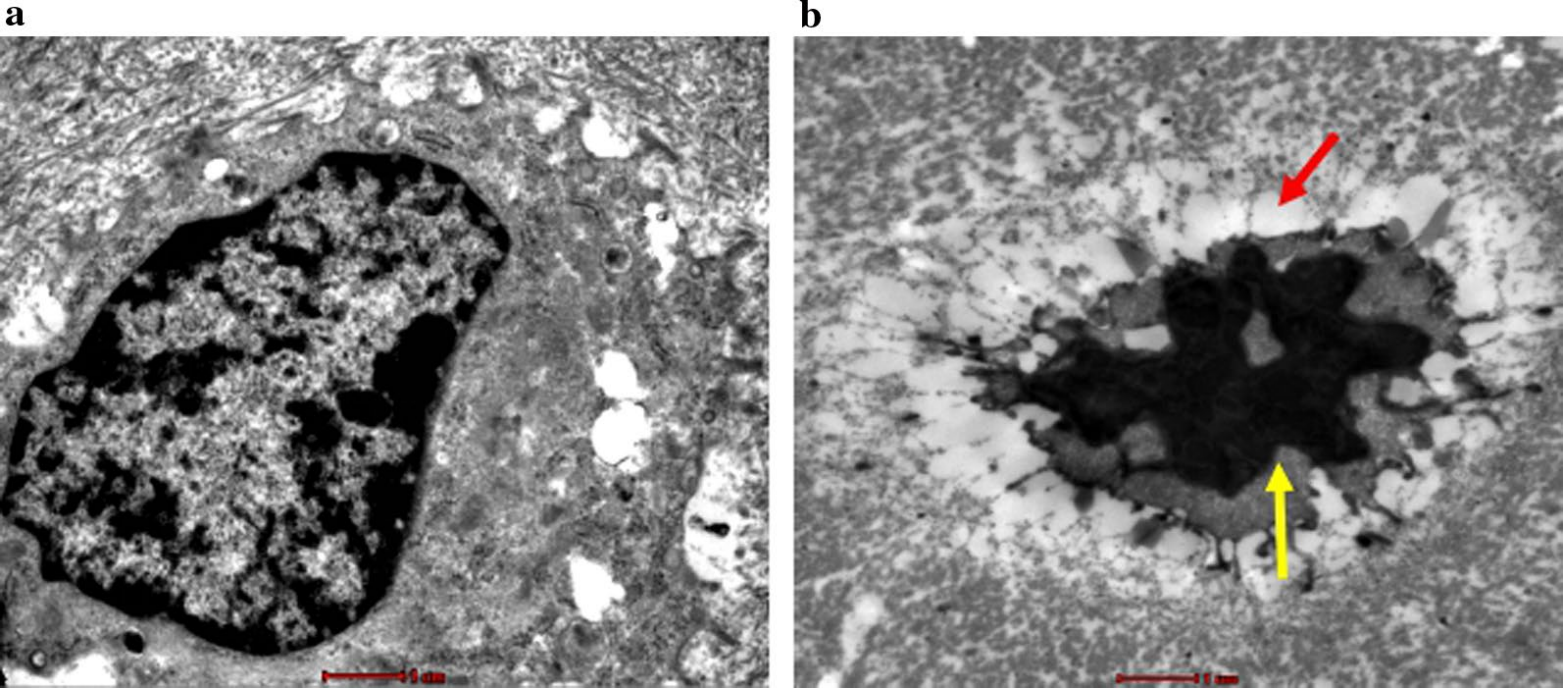
Morphological changes of apoptotic cells was detected via TEM (× 9900). Control group (images **a**): osteocyte was round or oval, large in size and rich in organelle, the chromatin was clear and uniform, and the nuclear membrane was intact. Model group showed typical apoptotic morphological features: the bone cell volume became smaller, the nucleus was pyknotic, the shape was irregular, the cell edge vacuolation, the nuclear chromatin was concentrated and marginalized (the red arrow), and the intracellular electron density was deepened, the heterochromatin was rich in the nucleus and distributed in large pieces (the black arrow). (images **b**). Scale bar = 2 um

The bone cells of the control group were round or oval, located in the bone lacuna, and the morphology of the bone lacuna was basically the same, and the nuclear membrane was intact. The nucleus was large, the nucleoli were visible, the chromatin was clear and uniform, the organelles in the cytoplasm were abundant, and the rough endoplasmic reticulum and mitochondria were abundant (Fig. 5a). From 2nd to 6th week, the model group showed that the bone cell volume became smaller, the nucleus was pyknotic, the shape was irregular, the cell edge was vacuolated, the nuclear chromatin was concentrated and marginalized, and the intracellular electron density was deepened. The heterochromatin was abundant and there were typical apoptotic morphological features such as large blocky distribution (Fig. 5b).

### Correlation analysis

**Fig. 6:**
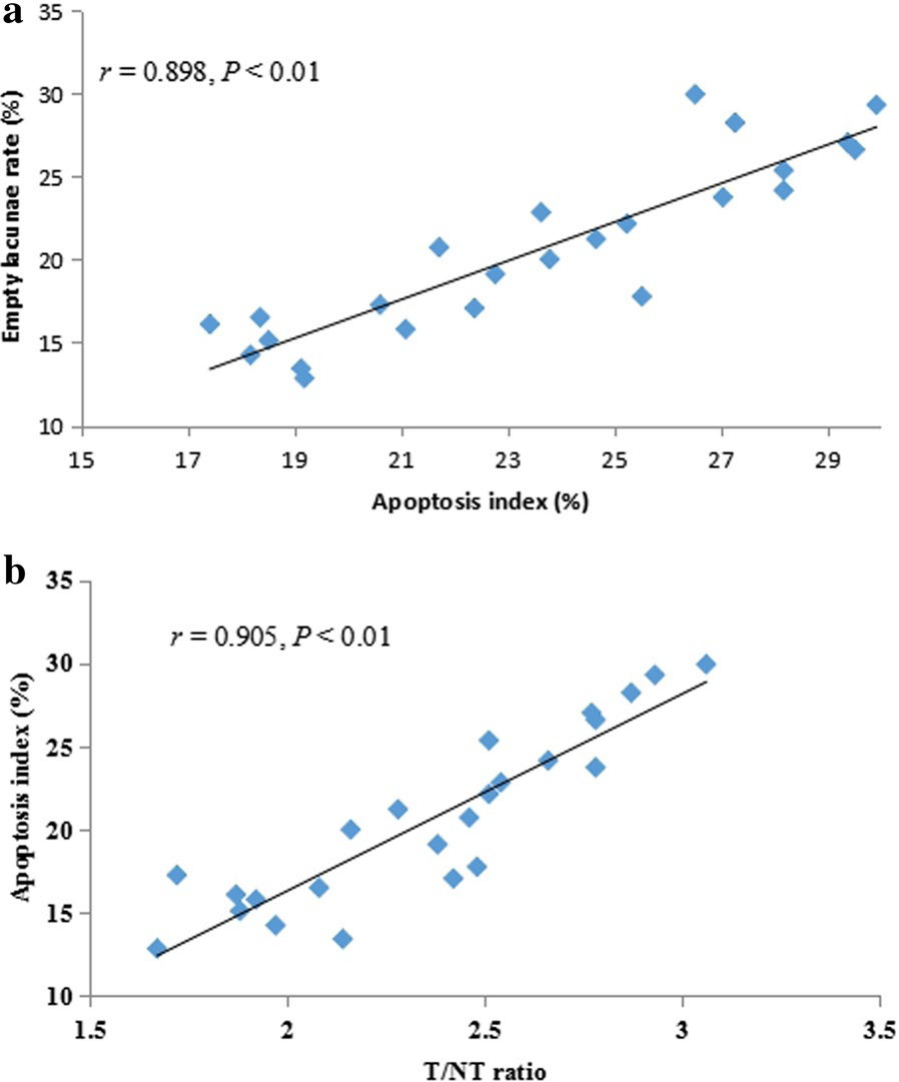
Pearson correlation analysis. Positive correlation between the empty lacunae rate and the apoptosis index (**a**), positive correlation analysis between the apoptosis index and the T/NT ratio (**b**). T/NT, target tissue and non-target tissue

The results of Pearson correlation analysis showed that the empty lacunae rate and the apoptosis index (Fig. 6a), and the apoptosis index and the T/NT ratio were significantly positively correlated at each time point in the model group (Fig. 6b) (*P* < 0.05), indicating that apoptosis is closely related to the occurrence and development of SIFHN, and that the T/NT ratio of the femoral head lesions has a significant correlation with the apoptosis index.

## Discussion

In this study, early animal models of femoral head necrosis was successfully established using the method of horse serum combined with hormone modeling. It was found that bone cell apoptosis was closely related to the occurrence and progression of SIFNH. Furthermore, we confirmed ^99m^Tc-Cys-Annexin V SPECT imaging could detect femoral head necrosis earlier than MRI in the animal models.

Because clinical SIFHN patients generally occur on the basis of the use of hormone-related diseases, such as SARS, systemic lupus erythematosus, rheumatoid arthritis, septic shock, nephrotic syndrome, leukemia, organ transplantation and other diseases, which have a common feature that the body already has a vasculature or an immune system disorder before applying hormones (Matsui et al. 1992). Therefore, in order to simulate the actual clinical situation of SIFNH in the establishment of animal models, we applied horse serum before applying hormones. The results showed that modeling success rate of hormone combined with horse serum method (84.62%) was significantly higher than that of hormone alone induction method (45.8%) (Tian et al. 2014), and the animal mortality rate (13.33%) by using hormone combined with horse serum method was significantly lower than that of the hormone combined with endotoxin modeling method (50%) (Liu et al. 2017).

Further, we used MRI (Qiang et al. 2015) and ARCO staging criteria (Gardeniers 1992) to verify whether it was the early SIFHN animal model. The results showed that there was no abnormality in conventional MRI in the model group at 2nd week after injection of hormones, and histopathological findings confirmed the osteonecrosis of nuclear pyknosis and empty lacunae, indicating that the model was in the early stage of femoral head necrosis. (ARCO stage 0). At 4th to 6th week after the injection of hormones, MRI showed an abnormal signal of osteonecrosis, and histopathology further confirmed that the diagnostic criteria of typical osteonecrosis with a significant increase in the number of empty bone lacuna in the subchondral area, indicating that the model was at early stage of SIFNH (ARCO stage I). Therefore, the early SIFHN animal model established was convenient for studying the pathogenesis and early diagnosis and treatment of femoral head necrosis.

Many studies (Youm et al. 2010) have found that excessive use of hormones can induce osteoblasts and bone cell apoptosis, and apoptosis gradually accumulates, eventually leading to necrosis of the femoral head. Apoptosis plays an important role in SIFHN (Bai et al. 2016; Wang et al. 2016; Qiang et al. 2015). In this study, TUNEL technology was used to detect apoptotic bone cells at 2nd week, and the apoptosis characteristics were more obvious at 4th–6th weeks, which confirmed the pathogenesis of apoptosis in SIFHN. A large number of DNA double-strand breaks are considered to be the most prominent feature of TUNEL detection of apoptosis (Fayzullina and Martin 2014), but other causes of DNA single-strand breaks can be detected as positive. For example, cell necrosis or autolysis can also make TUNEL positive. (Loo 2011), resulting in lower specificity and higher false positive rates. Therefore, in order to rule out the interference of cell necrosis, transmission electron microscopy was used to observe the typical morphological features of apoptosis. Transmission electron microscopy is a cell morphology examination to observe the ultrastructure of cells, and cell morphology observation is considered to be a gold standard method for identifying apoptotic cells (Huang et al. 2018). Therefore, the data we obtained are more objective and better reveal the intrinsic relationship between apoptosis and SIFNH.

^99m^Tc-Cys-Annexin V SPECT imaging is a non-invasive radionuclide imaging method that can dynamically monitor apoptosis. It can effectively avoid the invasiveness of in vitro apoptosis detection methods and difficult to observe dynamically. Lu (2013) results showed that ^99m^Tc-Cys-Annexin V was mainly excreted by the kidneys, and the liver, lung and bladder had high radioactivity concentration, while the uptake in bone and muscle was low. The results of this experiment also confirmed that it is feasible to detect the apoptosis of SIFHN by ^99m^Tc-Cys-Annexin V. However, our previous study (Wang et al. 2016) found that there were still some problems in the ^99m^Tc-Cys-Annexin V SPECT imaging. ^99m^Tc-Cys-Annexin was highly concentrated in the kidneys and bladder, and the rabbit femoral head was also very close to the bladder. If the model rabbit does not ban drinking water before imaging, rabbit bladder filling may cause high radioactivity in the bladder, which affected the imaging of bilateral femoral head necrosis, which ultimately leaded to misjudgment of the results. Therefore, in this study, we banned drinking water for model rabbits 8 h before SPECT imaging, and used infantile catheters to urinate to avoid false positives caused by bladder filling.

In the ultra-early stage of the lesion (ARCO stage 0), the qualitative and semi-quantitative methods of ^99m^Tc-Cys-Annexin V SPECT imaging can detect femoral head necrosis, while MRI has no abnormal signal of femoral head necrosis. In the early stage of the lesion (ARCO stage I), ^99m^Tc-Cys-Annexin V SPECT imaging and MRI both could detect abnormal signals of femoral head necrosis, indicating that ^99m^Tc-Cys-Annexin V SPECT imaging could detect femoral head necrosis earlier than MRI, which had the possibility of early or even ultra-early diagnosis of femoral head necrosis in rabbits. In addition, radionuclide imaging has advantages in several cases: early detection of SPECT in patients with femoral head necrosis after renal transplantation may be more sensitive than MRI (Ryu et al. 2002). In some cases of bone infection (Horger et al. 2007) and suspected bone metastases (Utsunomiya et al. 2006), SPECT may have a greater diagnostic contribution, and SPECT imaging may be used as an alternative test in the case of MRI examination contraindications. These results suggested that ^99m^Tc-Cys-Annexin V SPECT imaging was a reliable method for diagnosing early SIFHN based on the mechanism of apoptosis. Because there was a significant positive correlation between the empty lacunae rate and apoptosis index, as well as the apoptosis index and T / NT ratio at each time point in the model group, therefore, the degree of femoral head necrosis can be reflected by the concentration of imaging agent in the femoral head and the T/NT ratio of the imaging agent, ultimately providing the basis for the SIFHN staging. However, there are still some problems in this study: First, there is no ^99m^Tc-Cys-Annexin V SPECT experimental study on the anti-apoptotic intervention of early SIFHN. Second, still need to expand the sample size to reduce error. Third, compared with CT, MRI and ultrasound examinations, radionuclide imaging has lower resolution and poorer clarity, which affects the display of fine structures and the precise positioning of lesions, and it is necessary to appropriately select or combine various imaging examinations when confirming relevant diseases. Although the radionuclide imaging contrast agent is introduced into the body by intravenous injection, the adverse reaction rate is much lower than that of X-ray contrast agent, and the radiation absorbed dose caused by one inspection is very low, and it is mostly lower than that of conventional X-ray examination, but it is still radioactive and has health risks. In addition, compared with X-ray examinations and ultrasound examinations, radionuclide scanning imaging is the same as MRI, which requires a longer time and higher cost, and is rarely used in emergency patients.

## Conclusions

In summary, ^99m^Tc-Cys-Annexin V SPECT imaging, which can diagnose earlier stage of femoral head necrosis compared with MRI, can non-invasively diagnose the early stage of apoptosis in rabbit SIFHN model, which has the value in the diagnosis of early and even ultra-early stage femoral head necrosis.

## Data Availability

All data generated or analyzed during this study are included in this article.

## Abbreviations

SARS: Severe acute respiratory syndrome
SIFHN: Steroid-induced femoral head necrosis
ARCO: Association research circulation osseous
CT: Computed tomography
ECT: Emission computed tomography
MRI: Magnetic resonance imaging
SPECT: Single-photon emission computed tomography
ROI: Region of interest
T/NT: Target tissue and non-target tissue
HE: Hematoxylin–Eosin
TUNEL: Terminal deoxynucleotidyl transferase (TdT)-mediated dUTP nick end labeling
TEM: Transmission electron microscopy
EDTA: Ethylenediaminetetraacetic acid
